# Performance evaluation of respiratory motion‐synchronized dynamic IMRT delivery

**DOI:** 10.1120/jacmp.v14i3.4103

**Published:** 2013-05-06

**Authors:** S.A. Yoganathan, K.J. Maria Das, Arpita Agarwal, Shaleen Kumar

**Affiliations:** ^1^ Gautam Buddh Technical University Lucknow Uttar Pradesh India; ^2^ Department of Radiotherapy Sanjay Gandhi Postgraduate Institute of Medical Sciences Lucknow Uttar Pradesh India

**Keywords:** DMLC, IMRT, 4D, tracking, gating, dose rate

## Abstract

The purpose of this study was to evaluate the capabilities of DMLC to deliver the respiratory motion‐synchronized dynamic IMRT (MS‐IMRT) treatments under various dose rates. In order to create MS‐IMRT plans, the DMLC leaf motions in dynamic IMRT plans of eight lung patients were synchronized with the respiratory motion of breathing period 4 sec and amplitude 2 cm (peak to peak) using an in‐house developed leaf position modification program. The MS‐IMRT plans were generated for the dose rates of 100 MU/min, 400 MU/min, and 600 MU/min. All the MS‐IMRT plans were delivered in a medical linear accelerator, and the fluences were measured using a 2D ion chamber array, placed over a moving platform. The accuracy of MS‐IMRT deliveries was evaluated with respect to static deliveries (no compensation for target motion) using gamma test. In addition, the fluences of gated delivery of 30% duty cycle and non‐MS‐IMRT deliveries were also measured and compared with static deliveries. The MS‐IMRT was better in terms of dosimetric accuracy, compared to gated and non‐MS‐IMRT deliveries. The dosimetric accuracy was observed to be significantly better for 100 MU/min MS‐IMRT. However, the use of high‐dose rate in a MS‐IMRT delivery introduced dose‐rate modulation/beam hold‐offs that affected the synchronization between the DMLC leaf motion and target motion. This resulted in more dose deviations in MS‐IMRT deliveries at the dose rate of 600 MU/min.

PACS numbers: 87.53.kn, 87.56.N‐

## I. INTRODUCTION

Intrafractional motion caused by respiration is an important concern in upper abdomen and thoracic sites and it results geometric, as well as dosimetric, uncertainties in radiation therapy treatments.^1^, ^2^, ^3^, ^4^, ^5^ Advanced techniques, such as stereotactic body radiation therapy, utilizes delivery of higher dose to the target in a fewer fractions and this highlights the importance of managing these intrafractional uncertainties.^(6)^

Techniques such as active or passive breath‐hold^7^, ^8^, ^9^ and respiratory gating^10^, ^11^, ^12^ are commonly employed to account the intrafractional motion and reduce the internal margin in planning target volume. Breath‐hold technique cannot be applicable to all lung patients because it may not be tolerated by pulmonary‐compromised patients.^(13)^ Conversely, the gated technique prolongs the treatment time, which is not favorable especially in IMRT as it would compromise the tumor control probability.^14^, ^15^ Further details about the above techniques may be found in the literature.^(16)^

An alternate solution to account for the respiratory motion is to chase or track the moving target. Tracking of moving target can be achieved by various approaches (e.g., couch movement,^17^, ^18^ robotic linac,^19^, ^20^, ^21^ dynamic multileaf collimator (DMLC),^22^, ^23^, ^24^, ^25^, ^26^, ^27^, ^28^, ^29^, ^30^, ^31^, ^32^, ^33^, ^34^, ^35^, ^36^, ^37^ and gimbaled linac^38^, ^39^, ^40^, ^41^). Among these tracking methods, DMLC‐based target tracking was the focus of this work.

The DMLC‐based target tracking was first empirically demonstrated by Keall et al.^(22)^ who described the technique as motion adaptive X‐ray therapy (MAX‐T). It has been shown that the DMLC could be effectively used to manage the target motion with submillimeter geometric accuracy.^23^, ^24^ The DMLC was also investigated to deliver target motion‐synchronized intensity‐modulated radiation therapy (MS‐IMRT). Papiez et al.^29^, ^30^, ^31^ discussed algorithms for DMLC leaf‐pair optimal control IMRT delivery to moving and deforming targets. McQuaid and Webb,^(33)^ McClelland et al.,^(34)^ and Webb et al.^35^, ^36^, ^37^ demonstrated an approach to deliver the dynamic IMRT treatments to moving targets; these studies considered the regular, as well as differential, target motions.

In MS‐IMRT treatments, two methods are generally used to obtain the target motion. One utilizes the online/real‐time information^23^, ^25^ about the target position, and the other utilizes off‐line/prior information.^26^, ^27^ In the real‐time method, the target motion information is monitored during treatment delivery using external/internal surrogates, and the DMLC apertures are reshaped in real time to compensate for the online monitored target motion. In off‐line method, the target motion is measured at simulation stage and used to create the DMLC‐based motion‐synchronized deliveries; synchronized moving aperture radiation therapy (SMART)^(26)^ and breathing synchronized delivery (BSD)^(27)^ are two such techniques. During these treatment deliveries, this synchronization between the target motion and DMLC motion should be maintained. However, mechanical motion capabilities of DMLC leaf impose constraints for maintaining this synchronization in MS‐IMRT because the DMLC has not only to move for beam intensity modulation, but also has to follow the target motion.^(42)^ Hence, it is important to investigate the capabilities of DMLC to deliver the MS‐IMRT treatments, especially under various circumstances.

The purpose of this study was to evaluate the capabilities of DMLC to deliver the MS‐IMRT treatments under various dose rates (DR) using prior information of target motion. Furthermore, the dosimetric results of MS‐IMRT deliveries were also compared with gated and non‐MS‐IMRT (no compensation for target motion) deliveries. In this study, the target motion only along the DMLC motion was considered because the lung tumor motion is larger in the craniocaudal direction for lower‐lobe unfixed tumors.^(43)^

## II. MATERIALS AND METHODS

A DMLC leaf motion modification method was developed to incorporate the prior target motion due to respiration into a dynamic IMRT delivery. Using this method, the MS‐IMRT plans were created for eight lung patients who had previously been planned for dynamic IMRT. The dosimetric accuracy of the MS‐IMRT deliveries was evaluated for three dose rates: 100 MU/min, 400 MU/min, and 600 MU/min. Further, the dosimetric results of MS‐IMRT deliveries were compared with corresponding gated deliveries of duty cycle 30% and non‐MS‐IMRT deliveries.

### A. Respiratory motion‐synchronization method

**Figure 1:**
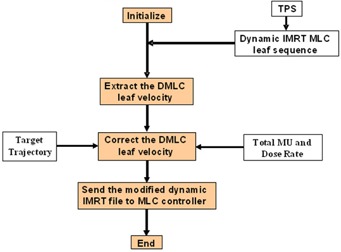
The work flow of respiratory motion synchronization in dynamic IMRT.

The process of respiratory motion‐synchronization in conformal delivery is relatively simple and straightforward. But, this process is complicated in dynamic IMRT since the DMLC leafs are already moving. In principle, the DMLC leafs should not only compensate the target motion, but also they should preserve the intended intensity pattern in a MS‐IMRT delivery. For this purpose, an automatic program was developed in MATLAB (ver. 7.0; The MathWorks, Natick, MA) and the workflow of this program is shown in Fig. 1. In this study, the target motion was incorporated into the DMLC leaf motions which was created after optimization process (i.e., the DMLC leaf motion file (actual fluence) calculated by leaf sequencing algorithm was used). The program requires two inputs: original DMLC leaf motion information (including dose rate and total monitor unit (MU)) of a dynamic IMRT plan, and the trajectory of the moving target. Using this information, the program superimposes the target trajectory into the original DMLC leaf motions.

The program corrects the individual DMLC leaf motions for the prior information of target motion. The respiratory motion‐corrected DMLC leaf velocity (${V_{MLC}}^{\prime }$) is determined as follows:

(1) VMLC'={VMLCfor VT=0VMLC+|VT|for VT>0VMLC‐|VT|for VT<0

The program extracts each DMLC leaf velocity ($V_{\text{MLC}}$) from the original DMLC leaf motion file and adds the velocity of the moving target ($V^{T}$) into it during the forward (positive) direction of target motion (i.e., both the target and DMLC are moving in the same direction). Conversely, the program subtracts the $V_{T}$ from the $V_{\text{MLC}}$ during backward (negative) direction of target motion (i.e., both the target and DMLC are moving in opposite direction). During the forward motion of the target, the DMLC has to move fast enough to compensate the target motion and, in addition, it has to preserve the required intensity modulation. Whereas, during the backward motion of the target, the DMLC has to move slow enough to compensate the target motion as well as preserve the required intensity modulation. During the backward target motion, the resultant direction of leaf motion is always towards the highest velocity value (i.e., if the DMLC leaf velocity is greater than target velocity, the resultant velocity after subtraction would be towards DMLC leaf motion). Similarly, if the target velocity is greater than DMLC leaf velocity, the resultant velocity after subtraction would be towards target motion.

### B. Designing motion‐synchronized delivery

The respiratory motion considered in this study was having ${\text{cos}}^{4}(t)$‐shaped motion in craniocaudal direction with a fixed amplitude of 2 cm peak to peak and the breathing period of 4 sec.^(44)^ Previously planned dynamic IMRT plans of eight lung patients were retrospectively included in this study. These IMRT plans were optimized using conventional (3D) CT images for a total dose of 70 Gy in 35 fractions with 6 MV photon beam at a dose rate of 400 MU/min in a commercial treatment planning system (Eclipse, Varian Medical Systems, Palo Alto, CA). The DMLC leaf motions were calculated using leaf motion calculator (LMC) (ver. 8.6) by keeping the effective limiting leaf velocity as 2.5 cm/sec. In order to keep the DMLC leaf motion along the target motion, the collimator rotation was fixed at 90° for all fields. These DMLC leaf motions of the dynamic IMRT plans along with MU and dose‐rate information were imported into the in‐house program. The velocity of the individual DMLC leafs was modified to incorporate the above assumed target motion. During motion synchronization, the initial respiratory phase was set as 320° which was determined as an optimal parameter.^(42)^ In a dynamic IMRT planning, variation in the dose rate alters the velocity of the DMLC leaf and MU required for delivering the treatment.^(45)^ Hence, dynamic IMRT plans with dose rate of 100 MU/min and 600 MU/min were also generated for each patient. The DMLC leaf motions of these plans were also imported into the program for motion synchronization. During the motion‐synchronization process, no attempt was made to restrict the DMLC leaf velocity below the effective limiting leaf velocity. Further, the dose rate was considered as constant during MS‐IMRT deliveries.

### C. Evaluation of motion‐synchronized deliveries

The experimental verification of MS‐IMRT deliveries was performed in a medical linear accelerator (CL2100CD, Varian Medical Systems) equipped with millennium 120 DMLC system.

Dosimetric accuracy (2D fluences) of the MS‐IMRT deliveries was measured using I'MatriXX 2D ion chamber array device (Scanditronix Wellhöfer, Freiberg, Germany) which was sandwiched between Perspex slab phantoms. This detector system was placed over the QUASAR motion platform (Modus Medical Devices Inc., London, Canada) which was programmed to move in the craniocaudal direction (along the direction of DMLC leaf motion) for the assumed target motion. During the MS‐IMRT delivery the treatment was started manually whenever the target was in synchronization with the DMLC leaf motion. The real‐time position management (RPM) system was used to make this manual synchronization (i.e., the ‘beam on’ was made at 320° respiratory phase by observing the breathing cycle in RPM software). The measurements were carried out for static, non‐MS‐IMRT, gated, and MS‐IMRT deliveries at the dose rates of 100 MU/min, 400 MU/min, and 600 MU/min for all patients. A total of 96 measurements were performed for eight patients, which consisted of 24 measurements for each static, non‐MS‐IMRT, gated, and MS‐IMRT deliveries. The dosimetric measurements were carried out for all patients using the following experimental setups:

1. Static IMRT delivery: The I'MatriXX remained static (no motion) and the original dynamic IMRT plans (no compensation for respiratory motion) were delivered. These measurements were considered as reference.
2. Non‐MS‐IMRT delivery: The I'MatriXX movement was simulated for the assumed target motion and the original dynamic IMRT plans (no compensation for respiratory motion) were delivered.
3. MS‐IMRT delivery: The I'MatriXX movement was simulated for the assumed target motion and the MS‐IMRT plans (compensated for respiratory motion) were delivered.
4. Gated delivery: The RPM system was used to deliver the gated treatments. A two dot passive marker block was placed on chest wall of the moving QUASAR phantom. An infrared camera tracks the movement of the markers to produce the respiratory motion of phantom. A gating widow of 30% duty cycle was set at end expiration of the wave. The original IMRT plans (no compensation for respiratory motion) were delivered on the moving detector under this gated operation.

The dosimetric measurements of non‐MS‐IMRT, gated IMRT, and MS‐IMRT deliveries were compared with static delivery using gamma evaluation^(46)^ criteria of 2% dose difference and 2 m distance to agreement. The percentage of pixels which had γ<1 was recorded for analysis. Statistical analyses were also performed using the Student's t‐test (two‐tailed, heteroscedastic). Differences were considered to be significant for p‐value <0.05. During the above measurements, the MLC controller was programmed to record the DynaLog (Varian Medical Systems) files for leaf positional error analysis.

## III. RESULTS

**Figure 2:**
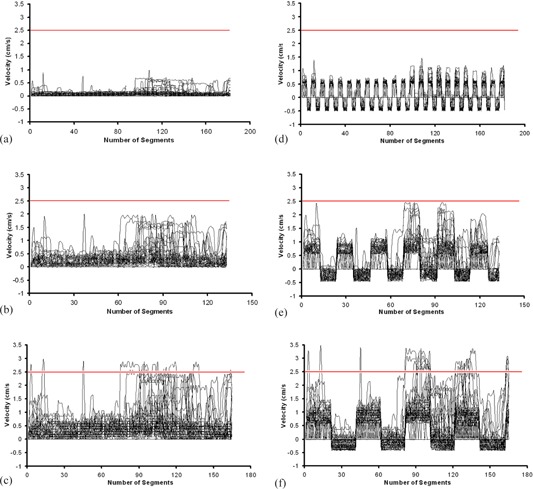
The velocities of 60 DMLC leafs in a original dynamic IMRT plan for the dose rates of 100 MU/min (a), 400 MU/min (b), and 600 MU/min (c) are shown, and demonstrating that the velocities of all leafs are positive. The velocities of DMLC leafs in MS‐IMRT plans for the dose rates of 100 MU/min (d), 400 MU/min (e), and 600 MU/min (f). In MS‐IMRT delivery, the DMLC leaf velocities obtain both positive and negative values to account forward and backward motion of target, and hence exceed the effective limiting leaf velocity (2.5 cm/sec) at 600 MU/min.

In order to deliver the intended dose to a moving target, the velocities of the DMLC leafs in the original IMRT plans were modified to synchronize the prior information of target motion. Figures 2(a), (b), and (c) show DMLC leaf velocities of original IMRT (non‐synchronized) plans of a single patient for the dose rates of 100 MU/min, 400 MU/min, and 600 MU/min. It is revealed from these figures that the velocities of DMLC leafs are increased as the dose rate is increased. Figures 2(d), (e), and (f) show DMLC leaf velocities of MS‐IMRT plans for the dose rates of 100 MU/min, 400 MU/min, and 600 MU/min. It is observed from these figures that the velocities of the DMLC leafs are modified (in positive and negative directions) to account the target motion. Further, the required velocity to deliver the MS‐IMRT plan exceeded the leaf velocity value 3.0 cm/sec for the dose rate of 600 MU/min in three (patients 3, 4 and 7) out of eight patients.

**Figure 3:**
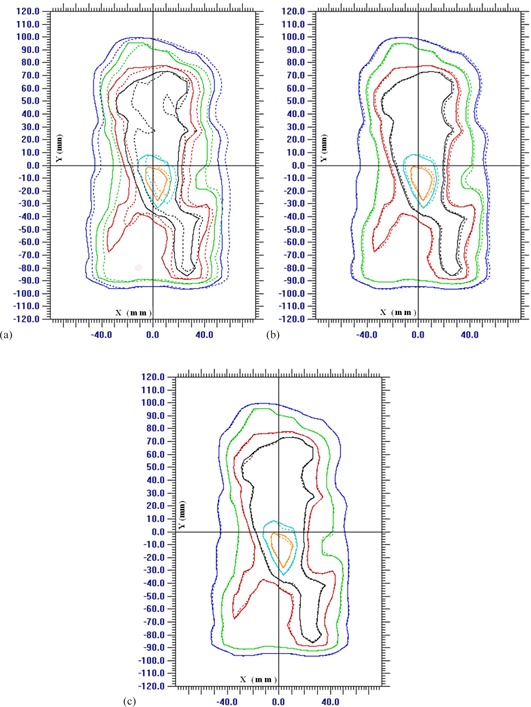
Isodose distributions (dashed lines) of non‐MS‐IMRT (a), gated IMRT (b), and MS‐IMRT (c) deliveries at the dose rate of 400 MU/min are compared with the corresponding static isodose distribution (solid lines). The assumed target movement was along the DMLC motion with 2 cm peak‐to‐peak amplitude and 4 sec breathing period. (Isodose lines: 95% = orange, 90% = cyan, 80% = black, 70% = red, 50% = green, and 30% = blue.)

**Figure 4:**
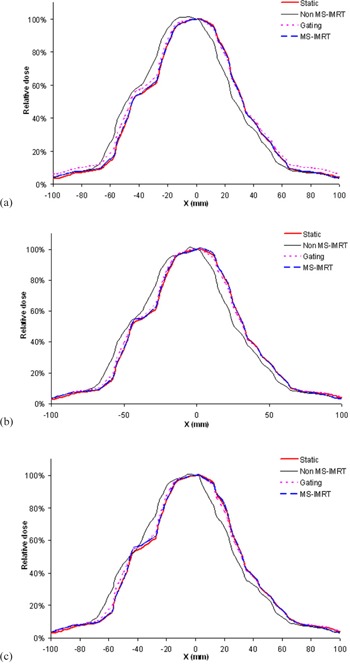
Dose profiles of various techniques are shown for dose rates 100 MU/min (a), 400 MU/min (b), and 600 MU/min (c). These profiles were obtained along the DMLC leaf motion direction at the central axis of Fig. 3.

The dose distribution of non‐MS‐IMRT delivery was compared with static reference delivery and is shown in Fig. 3(a) for a single patient at the dose rate of 400 MU/min. Similar comparisons were also performed for gated and MS‐IMRT deliveries and are shown in Figs. 3(b) and (c). Further to highlight the effect of dose rate, the dose profiles were extracted along the DMLC leaf motion direction across the Fig. 3 (x‐axis) and are shown for dose rates 100 MU/min, 400 MU/min, and 600 MU/min in Figs. 4(a), (b), and (c) for various techniques.

The percentage of pixels which passed the gamma criteria of 2% dose difference and 2 mm distance to agreement is shown in Table 1. In the absence of motion synchronization, the dose deviation with respect to static was larger, and the percentage of pixels passing the gamma test was in the range of 58.5 to 80.66 for the dose rate of 100 MU/min, 57.71 to 77.54 for 400 MU/min, and 53.42 to 75.1 for 600 MU/min. However, when the gating method was exploited, this dose deviation was significantly reduced and percentage of pixels passing the gamma test was in the range of 85.17 to 88.67 for 100 MU/min (gating vs. non‐MS‐IMRT; p<0.001), 82.62 to 92.97 for 400 MU/min (p<0.001), and 82.13 to 91.41 for 600 MU/min (p<0.001).

On the other hand, the MS‐IMRT resulted in further significant improvement in dose delivery compared with gated deliveries. In the MS‐IMRT delivery, the percentage of pixels passing the gamma test was in the range of 91.89 to 98.44 for 100 MU/min, 89.16 to 98.34 for 400 MU/min, and 77.73 to 96.48 for 600 MU/min.

The percentage deviation of pixels passing the gamma test for gated and non‐MS‐IMRT deliveries with respect to corresponding MS‐IMRT deliveries was also calculated. The dosimetric accuracy of MS‐IMRT deliveries was superior over non‐MS‐IMRT deliveries by (mean±SD)28.97±7.05%(p<0.001),29.85±6.49%(p<0.001), and 28.62±6.88%(p<0.001) for the dose rates of 100 MU/min, 400 MU/min, and 600 MU/min, respectively. Similarly, the dosimetric accuracy of MS‐IMRT deliveries was advantageous over gated deliveries by 8.38±1.88%(p<0.001),6.19±4.79%(p=0.003), and 0.63±9.42%(p=0.685) for the dose rates of 100 MU/min, 400 MU/min, and 600 MU/min, respectively.

While comparing the impact of various dose rates, the MS‐IMRT delivery with low dose rate (i.e., 100 MU/min) resulted in superior dosimetric accuracy compared with other dose rates (100 MU/min vs. 400 MU/min, p=0.252; 100 MU/min vs. 600 MU/min, p=0.027; and 400 MU/ min vs. 600 MU/min, p=0.095). It is observed that the MS‐IMRT delivery at a dose rate of 600 MU/min did not result in any additional benefit over corresponding gated delivery.

**Table 1:** Percentage of pixels passing the gamma criteria of 2% and 2 mm.

	*Dose Rate 100 MU/min*			*Dose Rate 400 MU/min*			*Dose Rate 600 MU/min*		
*Patient Number*	*Non‐MS‐IMRT*	*Gating*	*MS‐IMRT*	*Non‐MS‐IMRT*	*Gating*	*MS‐IMRT*	*Non‐MS‐IMRT*	*Gating*	*MS‐IMRT*
1	80.66	88.45	92.77	77.54	90.2	91.31	75.1	89.36	87.4
2	66.89	87.79	98.44	64.16	87.7	98.34	62.89	87.11	96.48
3	64.65	87.7	96.19	62.99	87.21	93.55	57.52	90.92	77.73
4	62.79	86.25	93.26	57.71	87.12	89.16	57.03	87.3	87.11
5	68.75	86.72	95.12	65.92	92.97	94.04	65.14	91.41	93.07
6	69.92	87.99	97.27	69.04	82.62	95.02	69.63	82.13	94.14
7	58.5	85.17	91.89	60.74	86.42	89.65	53.42	85.9	81.15
8	69.93	88.67	98.05	66.8	87.21	97.56	65.63	87.21	92.87
Average ± Standard Deviation	67.76±6.52	87.34±1.20	95.37±2.51	65.61±5.98	87.68±2.98	93.58±3.40	63.30±7.16	87.67±2.97	88.74±6.63

## IV. DISCUSSION

A leaf motion modification method was developed to synchronize the prior information of target motion into the delivery of dynamic IMRT. The accuracy of MS‐IMRT delivery was evaluated for the dose rates of 100 MU/min, 400 MU/min, and 600 MU/min in eight clinical patients. Further, the dosimetric results of MS‐IMRT deliveries were also compared with the corresponding gated (duty cycle 30%) and non‐MS‐IMRT deliveries.

The MS‐IMRT could deliver the intended dose to a moving target with superior dosimetric accuracy compared with gating and non‐MS‐IMRT techniques (Table 1). Since, the gating method had residual motion within the 30% gating window, it had slightly larger deviations compared with MS‐IMRT delivery. This residual motion is directly proportional to the gating window size or duty cycle. Hence, use of larger duty cycle will further degrade the delivered dose. The treatment efficiency of MS‐IMRT deliveries was nearly 100% for all patients (due to prior target motion synchronization); whereas, the gated delivery had only 30%. Hence, MS‐IMRT delivery was found to be superior in terms of dosimetric accuracy and treatment efficiency.

While comparing the dosimetric results of MS‐IMRT delivery for various dose rates, larger deviations were observed at high‐dose‐rate delivery. Because, the use of high‐dose rate in a dynamic IMRT delivery increased the DMLC leaf velocities (see Figs. 2(a), 2(b), b(2)). Further, the motion‐synchronization process resulted in addition of target velocity into the original DMLC leaf motion during the forward motion of target (Figs. 2(d), 2(e), e(2)). This higher DMLC leaf velocity resulted in larger dose deviation in the MS‐IMRT delivery.

In principle, LMC usually requires one of the DMLC leaves of a pair to be moving at maximum physical leaf velocity to minimize the treatment time and transmitted dose. But, the communication time delay between the control systems of MLC and accelerator imposes that the maximum leaf velocity used in a delivery should not be the maximum physical velocity limitation of DMLC, but rather the largest velocity which will not put the leaves out of tolerance with in the communication delay. This suggested the replacement of the maximum physical velocity of DMLC by the effective limiting leaf velocity.^(47)^ Hence, we used the effective limiting leaf velocity 2.5 cm/sec (not the maximum velocity) in our study. However, the synchronization of target motion resulted in the DMLC leaf velocity to exceed the effective limiting leaf velocity, especially at 600 MU/min. And even sometimes, the DMLC leaf motion exceeded the velocity value 3.0 cm/sec. The MS‐IMRT deliveries with prior knowledge of target motion always assume a constant dose rate.^(41)^ But, whenever the DMLC leaf motion exceeds the effective limiting leaf velocity, the MLC controller modulates the dose rate or holds the beam. This dose‐rate modulation/beam hold‐off would result in desynchronization of DMLC leaf motion with respect to target motion and, hence, wrong delivery to a moving target.

**Figure 5:**
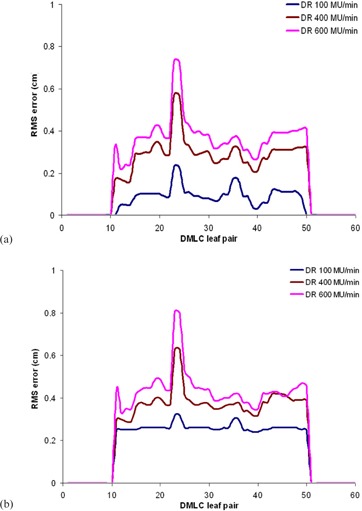
The leaf positional errors of DMLC leaf pairs recorded by DynaLog files are shown for static (a) and MS‐IMRT (b) delivery at the dose rates of 100 MU/min, 400 MU/min, and 600 MU/min.

Further, apart from dose‐rate modulation/beam hold‐offs, the increased DMLC leaf velocity resulted in more leaf positional errors during delivery.^(48)^ Data from the DynaLog files created from each delivery revealed that the leaf positional errors were consistently larger for higher leaf velocity deliveries (i.e., high‐dose‐rate deliveries). Figures 5(a) and (b) show the leaf positional errors in the DynaLog file recorded by MLC controller for static and MS‐IMRT deliveries at the dose rates of 100 MU/min, 400 MU/min, and 600 MU/min.

For the above reasons, we observed inferior dosimetric accuracy in MS‐IMRT deliveries at the dose rate of 600 MU/min. In addition, the MS‐IMRT deliveries at the dose rate of 600 MU/min did not result in any additional benefit over corresponding gated deliveries in terms of dosimetric accuracy. Therefore, in order to have better dose delivery in MS‐IMRT treatments, optimal dose rate should be used. It is observed from the Table 1 that the percentage of pixels passing the gamma criteria in MS‐IMRT delivery was roughly identical for dose rates of 100 MU/min and 400 MU/min (p=0.252), whereas 600 MU/min had larger (p=0.027) dose deviations. Hence, the dose rate of 300–400 MU/min may be utilized in a MS‐IMRT delivery, which will not compromise the dosimetric accuracy or treatment time.

In our study, we used the effective limiting leaf velocity as 2.5 cm/sec. Hence, in high‐dose‐rate IMRT plans, the DMLC leaf motions were already having the velocity in the range of 2.5 cm/sec. Therefore, whenever the motion‐synchronization process is attempted in a DMLC leaf motion file for high‐dose rate, the effective limiting leaf velocity should be reduced from the default value 2.5 cm/sec. Though the reduction of effective limiting leaf velocity increases the total MU in a dynamic IMRT delivery,^(45)^ it can be delivered with superior dosimetric accuracy to a moving target without dose‐rate modulation/beam hold‐offs. Hence, appropriate effective limiting leaf velocity and dose‐rate combination should be used in a MS‐IMRT delivery.

However, even after reducing the effective limiting leaf velocity, in some circumstances, the required DMLC velocity for a MS‐IMRT delivery may be above the physical DMLC velocity limitation. During these circumstances, one can utilize the following strategy. The program used in this study adds the target velocity into the DMLC leaf motion during the forward motion of target and subtracts it while the target is in backward motion. Only the addition of target velocity would result in the DMLC leaf motion exceeding the limit. Hence, in a motion‐synchronization process, whenever the DMLC leaf motion exceeds the maximum leaf velocity, we can introduce a beam ‘hold’ until the target moves backward; this would result in subtraction of target velocity from the original DMLC leaf motions and enable the DMLC leaf motions not to exceed the maximum DMLC leaf velocity. Although this process would increase the treatment time (by a factor of 2), the dosimetric accuracy in MS‐IMRT delivery would be ensured at high‐dose rates.

Results of this study about the impact of dose rate in a MS‐IMRT delivery agree with the earlier investigations by Xu et al.^(42)^ They systematically evaluated various clinical parameters such as collimator angle, initial phase, dose rate, and machine tolerance which affect the synchronization between the target motion and DMLC motion in SMART delivery. They determined the optimal clinical parameters to deliver the SMART treatment: collimator angle in the range from 90° to 110°, initial respiration phase 320°, appropriate dose rate, and MLC leaf tolerance. They included only one patient in their investigations. But, the performance of MS‐IMRT delivery would depend upon the complexity of the fluence to be delivered. Hence, we studied the impact of dose rate in MS‐IMRT delivery on cohort of eight patients. Among the eight patients, patient number 4 and 7 were having complex fluence patterns, therefore gamma pass rate in these patients were less compared to other patients. The impact of collimator rotation in MS‐IMRT delivery was not included in our study because we considered target motion only along the DMLC leaf motion direction. In addition, the optimal values were used for initial respiratory phase as 320° and the MLC leaf positional tolerance as 0.2 cm.

The presented results in this study are specific to the target motion of velocity ±0.5 cm/sec which was in the direction along the DMLC leaf motion. However, use of three dimensional target motions with larger velocity values would increase the DMLC leaf velocities further in a MS‐IMRT delivery. Hence, this would have a greater affect on the performance of delivery.

## V. CONCLUSIONS

The performance of MS‐IMRT delivery was evaluated for various dose rates and compared with gated and non‐MS‐IMRT deliveries. The dosimetric accuracy of MS‐IMRT deliveries was observed to be superior to both gated and non‐MS‐IMRT deliveries. The MS‐IMRT treatments were most reliably delivered when the dose rate was set to low values. And the use of high‐dose rate in MS‐IMRT delivery introduced dose‐rate modulation/beam hold‐offs, which affected the synchronization between the DMLC leaf motion and target motion. Hence, appropriate dose rate should be selected in MS‐IMRT deliveries. Strategies were also discussed to deal with the circumstances whenever the DMLC leaf motion exceeded the velocity constraints.

## ACKNOWLEDGMENTS

The authors would like to acknowledgment assistance received from the Department of Science & Technology, Grant No: IR/SO/LS 02/2003, Government of India.
